# *Pseudidiomarina piscicola* sp. nov., isolated from cultured European seabass, *Dicenthrarchus labrax*

**DOI:** 10.1007/s00203-020-02131-3

**Published:** 2020-12-07

**Authors:** M. Carmen Macián, Teresa Lucena, David R. Arahal, María A. Ruvira, Rosa Aznar, María J. Pujalte

**Affiliations:** Departamento de Microbiología y Ecología and Colección Española de Cultivos Tipo (CECT), Universitat de València, València, Spain

**Keywords:** *Pseudidiomarina*, *Idiomarina*, *Idiomarinaceae*, *Dicenthrarchus labrax*, Taxogenomics

## Abstract

Strain CECT 9734^ T^, a Gram-negative, aerobic, chemoorganotrophic bacterium, motile by polar flagella, was isolated from cultured European seabass, *Dicenthrarchus labrax,* in Spain. It grows from 5 to 42 ºC, 6–9 pH and 1–12% total salinity. Major cellular fatty acids are C_15:0_ iso, summed feature 9 (C_17:1_ iso *w9c*/C_16:0_ 10-methyl) and C_17:0_ iso. The genome size is 2.5 Mbp and G + C content is 49.5 mol%. Comparative analysis of the 16S rRNA gene sequence shows that the strain is a member of *Pseudidiomarina*, with highest similarities with *Pseudidiomarina halophila* (97.0%) and *Pseudidiomarina salinarum* (96.9%). Phylogenomic tree based on UBCG program shows *P. halophila* as its closest relative. ANI and in-silico DDH with other *Pseudidiomarina* spp. are lower than 87 and 20%, respectively, suggesting that strain CECT 9734^ T^ represents a new species, for which we propose the name *Pseudidiomarina piscicola* sp. nov. and CECT 9734^ T^ (= LUBLD50 7a^T^ = LMG 31044^ T^) as type strain.

**Supplementary Information:**

The online version contains supplementary material available at 10.1007/s00203-020-02131-3.

## Introduction

*Pseudidiomarina* is a genus belonging to the family *Idiomarinaceae*, proposed by Jean et al*.* 2006. In the following years, several species were described as members of the genus, including the reclassification of two species of *Idiomarina*. As more species were incorporated, more difficult was their classification, and finally, Taborda et al*.* (2009) based on the absence of distinguishing phenotypic characteristics, transferred the nine validly named species of the genus *Pseudidiomarina* to the genus *Idiomarina.* Recently, Liu et al. (2018) performed a wide genome-based study of the family *Idiomarinaceae*, including the genus *Aliidiomarina* (Huang et al. 2012), and proposed the genus *Pseudidiomarina* to be restored, establishing a taxonomic reclassification of many species in the family based on genomic analysis. As currently defined, *Pseudidiomarina* contains sixteen validly named species as stated in https://lpsn.dsmz.de/genus/pseudidiomarina (Parte 2018). One of them, *P. maritima*, has been recently revealed as a later heterotypic synonym of *P. tainanensis* (Liu et al. 2020).

*Pseudidiomarina* species comprise Gram-negative rods, strictly aerobic and chemoorganotrophic, mesophilic and halophilic which are isolated from seawater and sediments, solar salterns and saline lakes. Only one species, *P. aquimaris*, was isolated from a marine organism (the coral *Isopora pallifera*; Chen et al. 2012). Here we address the taxonomic characterization of strain CECT 9734^ T^, obtained from the liver of a cultured European seabass specimen, as a basis for the description of a new species of *Pseudidiomarina*, the first isolated from fish.

## Materials and methods

### Strain isolation and maintenance

Strain CECT 9734^ T^ was isolated from a cultured European seabass, *Dicenthrarchus labrax,* in Instituto de Acuicultura Torre de la Sal – Consejo Superior de Investigaciones Científicas (IATS-CSIC), Castellón, Spain (40°08′16.9"N 0°09′56.0"E) on May, 2000. This strain, originally labeled LUBLD50 7a, was obtained from a Marine Agar (MA) plate inoculated with a loop from the liver of a fish specimen and incubated at 26 ºC for 2 days. Once isolated, the strain was maintained as a stab culture in a semisolid Marine Agar screw-capped tube at room temperature in the dark. It was recovered in 2018, and deposited in the Spanish Type Culture Collection (CECT), where it is maintained by freeze-drying under the accession number CECT 9734^ T^. A partial 16S rRNA sequence suggested the strain belonged to the genus *Pseudidiomarina* and might be representative of a new species.

### Phenotypic characterization

Phenotypic characterization of strain CECT 9734^ T^ was performed following already described techniques (Pujalte et al. 2018). Cell morphology and motility was determined on wet mounts prepared from 48 h MA cultures of the strain, using phase contrast microscopy in a Leica DMRB fluorescence microscopy. Colonial morphology was observed in cultures on MA plates after 48 h of incubation. Temperature range for growth were determined on MA at 5, 15, 26, 30, 37, 42 and 45 ºC incubated up to 7 days (5 ºC were incubated up to 21 days). Salinity range was determined on MA diluted to give the desired salinity and supplemented with tryptone and yeast extract to restore original nutritional content (for salinities lower than 3%) or MA supplemented with NaCl as to achieve 6–20% (w/v) (for higher salinities). Hydrolytic activities on starch, casein, Tween-80 and alginate were determined on solid medium as already described (Pujalte et al. 2018). Oxidase test was performed with Oxoid oxidase strips and catalase was tested with 3% (v/v) H_2_O_2_. API ZYM, API 20NE and API 50 CH/E were used to determine enzymatic, assimilative and fermentative profiles, after supplementation of inoculating fluids with Marine Cations Supplement (MCS, Farmer and Hickmann-Brenner 2006). Sole carbon and energy sources used for growth were tested as described by Baumann and Baumann (1981).

Fatty acid methyl esters were extracted from cells grown on MA for 48 h at 26 ºC, and following the standard protocols described for the MIDI Microbial Identification System (Sasser 1990). Analysis of the cellular fatty acid content was performed at CECT using the Microbial Identification Sherlock software.

### 16S rRNA gene sequencing and phylogenetic analysis

An almost complete 16S rRNA sequence was obtained by PCR amplification and subsequent Sanger sequencing (Arahal et al. 2008), and compared by BLAST, identifying them as belonging to the genus *Pseudidiomarina*. Moreover, 16S rRNA sequence of strain CECT 9734^ T^ was annotated in its genome (1549 nt) (see below), and compared with the sequence obtained after amplification, resulting on a 100% coincidence. Phylogenetic analysis using different treeing methods were done using ARB (Ludwig et al. 2004).

### Genome sequencing and analysis

Genomic DNA was isolated using Gena Bioscience (Diffractia) following the standard protocol recommended by the manufacturer. Genome sequencing of strain CECT 9734^ T^, was achieved at Central Service of Support to Experimental Research (SCSIE) of the University of Valencia (Valencia, Spain) using an Illumina Miseq technology with 2 × 250 paired-end reads. The reads were analyzed for quality control using FASTQC, a common quality control tool developed by Babraham Bioinformatics to check raw sequencing data. After filtering, the remaining reads were assembled using SPAdes 3.9.0 software (Nurk et al. 2013). A plot, coverage versus length of the contigs, was performed to help in the choice of the parameters for contigs filtering. After the filtration of contigs (500 base pair (bp) length and 10–50 × kmer coverage). The bioinformatic tool CheckM v1.0.7 (Parks et al. 2015) was used to assess the genome quality prior to annotation using Prokka v1.12 (Seeman 2014) and RAST v2.0 (Rapid Annotation using Subsystem Technology) (Overbek et al*.* 2014). The process of quality assessment of reads, read-processing, assembly and annotation with Prokka was carried out in Linux OS, other tools were accessed online. The minimal standards for the quality of genome sequences and how they can be applied for taxonomic purposes (Chun et al. 2018) have been observed in this study.

Similarity between genomes was established using in-silico DNA–DNA Hybridization (*is*DDH) with the Genome-to-Genome Distance Calculator (GGDC 2.0) (Meier-Kolthoff et al. 2013) and Average Nucleotide Identity (ANI) index (Richter et al. 2015). Phylogenomic analysis was performed with UBCG (Up-to-date Bacterial Core Gene) using both nucleotide and aminoacidic sequences. UBCG is based on the analysis of 92 universal bacterial core gene sequences (Na et al. 2018).

## Results and discussion

Morphological, biochemical, and physiological traits of strain CECT 9734^ T^ are detailed in the species description and appear in Table 2 and in the Supplementary Table 2.

**Table 1:** In silico DNA–DNA hybridization (*is*DDH) values and Average Nucleotide Identify (ANI) index relating *P. piscicola* CECT 9734^ T^ to *Pseudidiomarina* species with available genomes

Species- strain	*is* DDH	ANIm
*P. salinarum* ISL-52^ T^	17.7	84.4
*P. aquimaris* SW15^T^	18.0	85.0
*P. sediminum* DSM 21906^ T^	18.2	84.7
*P. homiensis* PO-M2^T^	19.0	84.3
*P. planktonica* TS-T11^T^	17.5	83.9
*P. aestuarii* KYW314^T^	18.6	84.8
*P. taiwanensis* PIT1^T^	18.9	86.1
*P. halophila* BH195^T^	18.9	83.1
*P. insulisalsae* CVS-6^ T^	17.6	83.6
*P. indica* CGMCC 1.10824^ T^	18.8	85.2
*P. donghaiensis* 908033^ T^	19.4	86.3
*P. woesei* DSM 27808^ T^	19.3	86.2
*P. tainanensis* PIN1^T^	19.2	85.2

Two colony types were frequently seen, one transparent and other opaque, of the same size and form. Both colony types were submitted to 16S RNA gene sequencing and resulted identical.

Strain showed low reactivity: it did not grow on any carbon and energy source tested on Basal Medium Agar and it was not able to oxidize or ferment any carbohydrate in API 50 CH/E, even though the inoculation fluid was supplemented with marine salts Detailed results are shown in the species description.

Strain CECT 9734^ T^ grows in the presence of marine salts, it did not grow on media supplemented only with NaCl. Salinity range was 1–12% (on Marine Agar diluted / supplemented with NaCl); growth at 15% salinity was scarce and no growth was obtained at 0.5% or 18% or higher salinity. The temperature range for growth was 5–42 ºC (26–30 ºC optimum) and pH range was 6–9 (optimum: 7–8). No growth was obtained at pH 5.0 or less.

Major fatty acids were C_15:0_ iso (21.6%), Summed Feature 9 (C_17:1_ iso *ω9c*/ C_16:0_ 10-methyl) (18.5%) and C_17:0_ iso (14.8%), with minor amounts of C_16:0_ (9.0%), Summed Feature 3 (C_16:1_ *ω7c/ω6c*) (6.9%), C_11:0_ iso 3OH (5.1%) and Summed Feature 8 (C_18:1_ *ω7c/ω6c*) (4.9%) (Supplementary Table S1).

**Fig. 1:**
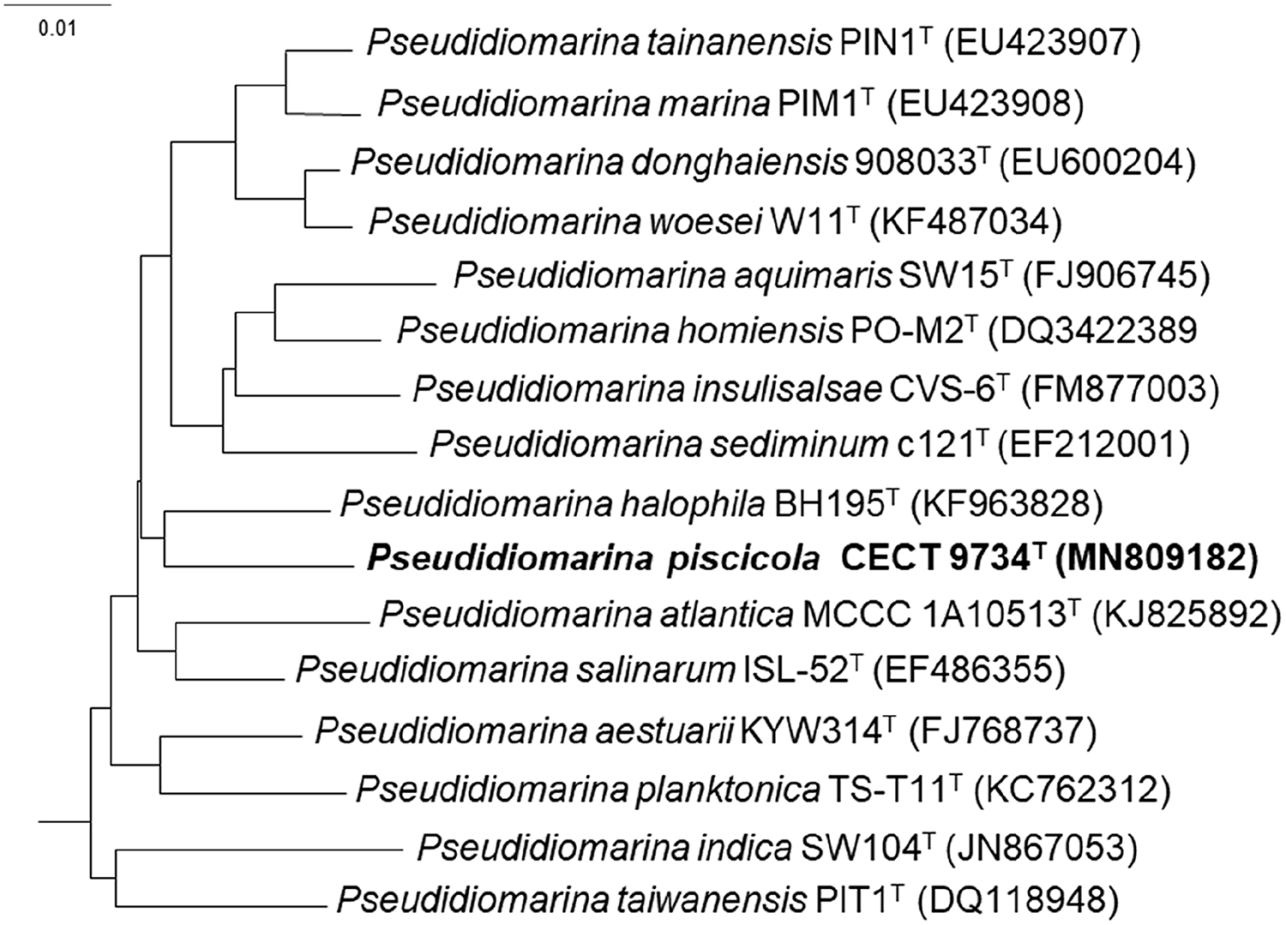
Phylogenetic reconstruction based on the 16S rRNA gene using the Neighbor joining method. Sequence accession numbers are given in parentheses. Bar indicates number of substitutions per position

The comparison of the 16S rRNA genome derived gene sequence with those of the type strains in the genus *Pseudidiomarina* using EzBioCloud identification tool (Yoon et al. 2017) showed that strain CECT 9734^ T^ was related to *Pseudidiomarina halophila* (97.0% sequence similarity), *P. salinarum* (96.9%), *P. homiensis* (96.7%) and *P. aquimaris* (96.7%). Phylogenetic tree based on 16S rRNA gene sequences, constructed using Neighbor Joining (NJ) method, show strain CECT 9734^ T^ within the species of the genus *Pseudidiomarina* (Fig. 1), with *P. halophila* as its closest relative.

The draft genome of strain CECT 9734^ T^ has an estimated size of 2.5 Mb, similar to the sizes found among *Idiomarinaceae* species, which are always below 3.1 Mb (Liu et al. 2018). The G + C content of the genome is 49.5 mol%. It is composed of 16 contigs with a N50 value of 441,728 nucleotides and final assembly coverage of 259 × . CheckM results of contamination and completeness were 0.0–98.99%, respectively. It contains 2360 protein coding sequences and 54 RNA genes, including a single rRNA operon and 51 tRNAs.

The annotated genome of CECT 9734^ T^ contains a gene coding for octaprenyl diphosphate synthase, suggesting that it is able to synthetize ubiquinone 8 (Q8), the most abundant respiratory quinone in the family *Idiomarinaceae*. It also contains genes coding for cardiolipin synthase, the enzyme responsible for diphosphatidyl glycerol (DPG) production and phosphatidylserine decarboxylase, which allows synthesis of phosphatidyl ethanolamine (PE). Along with phospahtidyl glycerol (PG), these are the major identified polar lipids of the genus *Pseudidiomarina*.

Although the strain was originally isolated from the liver of a fish, the genome of CECT 9734^ T^ does not contain any virulence or disease-related genes, and none of the protein secretion systems that have been related to pathogenicity is present in its genome. Siderophores are also absent. Eleven genes of a type IV pilus are present as well as multidrug resistance efflux pump component genes and several genes involved in copper, zinc, cobalt, cadmium and mercury resistance.

**Fig. 2:**
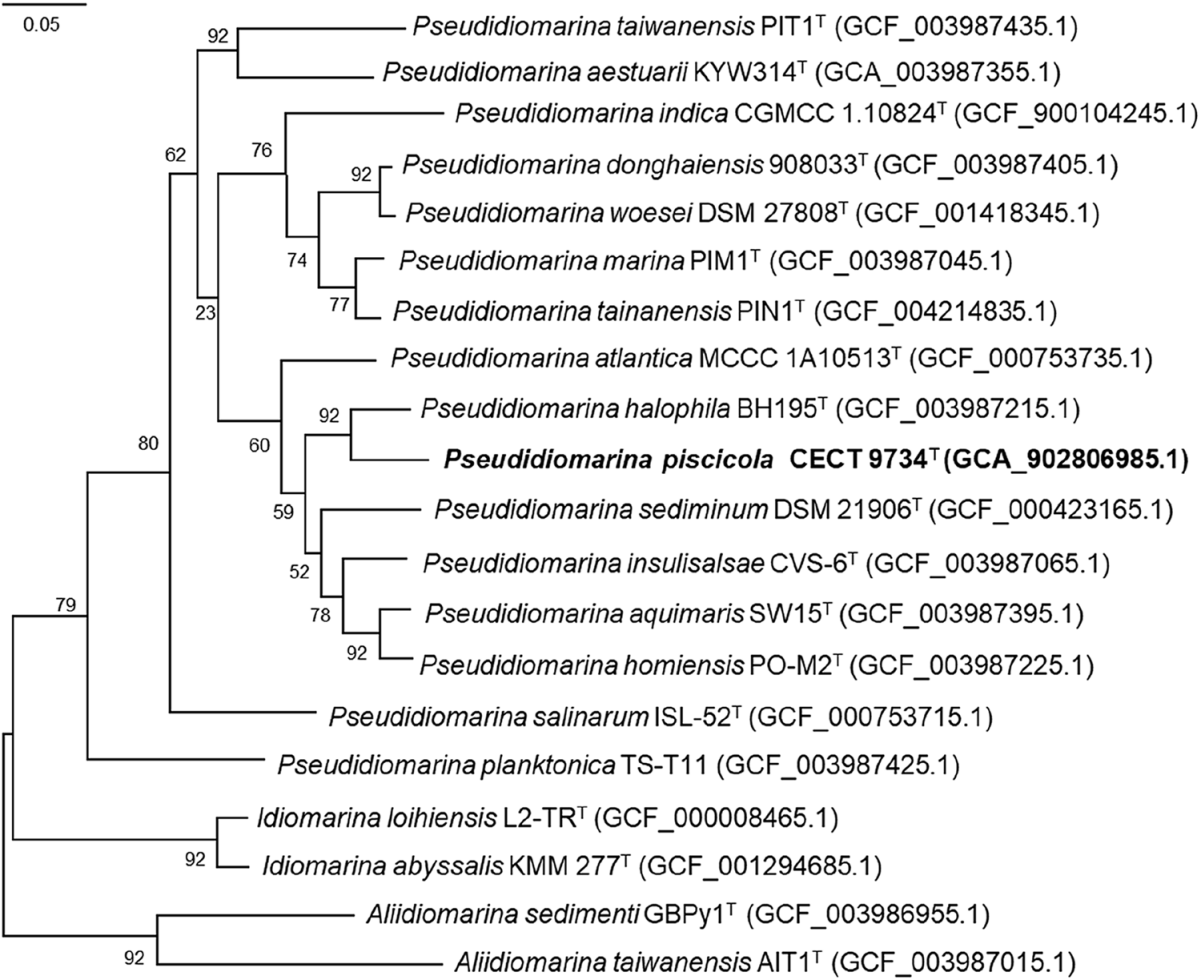
Phylogenomic tree generated with UBCG (Na et al., 2018) using aminoacids sequences. The numbers at the nodes indicate the Gene Support Index (GSI, maximal value is 92). Genome accession numbers are indicated in parentheses. Bar, 0.05 substitutions per position

Genome of strain CECT 9734^ T^ is related to their closer relatives by ANI values of less than 87% and in-silico DDH values lower than 20% (Table 1); both values are below the established cut-off figures for prokaryotic species delineation based on genomic data. UBCG-based phylogenomic trees confirm the relationships already displayed in the 16S rRNA gene based tree, with CECT 9734^ T^ as a part of the genus *Pseudidiomarina* and *P. halophila* as its closer relative (Fig. 2, Supplementary Figure S2). The three genera conforming the family are clearly defined both in the genomic nucleotide- and aminoacid-based trees, in complete agreement with the findings of Liu et al. 2018. A recently described species, *Pseudidiomarina gelatinasegens* (Li et al. 2020) was not included in the trees, but the similarity of its 16S rRNA gene to the one of CECT 9734^ T^ is lower enough (96.0%) to preclude species level relationships between them.

Thus, both phylogenetic and genomic information point to the novelty of CECT 9734^ T^ as representative of a yet undescribed *Pseudidiomarina* species.

Finally, from a phenotypic point of view, the strain displays the main characteristics of genus *Pseudidiomarina* (as emended by Liu et al. 2018). On the other hand, the strain displays several phenotypic traits that allow discrimination of CECT 9734^ T^ from other species in the genus. These differences affect pH range, urea, esculin and gelatin hydrolysis and enzymes acting on lipids, polypeptides and carbohydrates. Table 2 shows characteristics differentiating CECT 9734^ T^ from the two species most similar in 16S rRNA gene sequence.

**Table 2:** Differential characteristics between *Pseudidiomarina piscicola* CECT 9734^ T^ and its closest relatives

	1	2	3
Motility	+	−	+
pH range	6.0–9.0	3.5–10.5	6.0–8.0
Urea hydrolysis	−	+	−
Gelatin hydrolysis	+	+	−
Esculin hydrolysis	−	+	−
Enzymatic activities on API ZYM			
Estearase (C4), Esterase lipase (C8), Trypsin, α-Chymotrypsin	−	+	+
Valine arylamidase, Cystine arylamidase, Naphthol-AS-BI-phosphohydrolase, β-glucosidase	−	+	−
α-mannosidase	−	−	+
Acid from (API 50CH)			
d-fructose, amygdalin, arbutin, cellobiose, sucrose, trehalose	−	+	−

1: *P. piscicola* sp. nov. CECT 9734^ T^ (this study); 2: *P. halophila* BH195^T^ (Jean et al. 2006); 3: *P. salinarum* ISL-52^ T^ (Yoon et al. 2007). + : positive;—negative. All strains are positive for alkaline phosphatase and leucine aryl amidase. All strains are negative for acidic phosphatase, lipase (C14), α-galactosidase, β-galactosidase, β-glucuronidase, α-glucosidase, N-acetyl- β-glucosaminidase, α-fucosidase

In consequence, we propose to recognize the strain as representative of a new, yet unnamed species of the genus *Pseudidiomarina*, with the name of *Pseudidiomarina piscicola* sp. nov. and CECT 9734^ T^ (= LUBLD50 7a^T^ = LMG 31044^ T^) as the type strain of the species.

### Description of *Pseudidiomarina piscicola* sp. nov.

*Pseudidiomarina piscicola* (pis.ci’co.la. L. n. piscis fish; L. suff. -cola dweller; N.L. n. piscicola, fish -dweller).

Cells are Gram-reaction negative rods, motile by a polar flagellum. Colonies on Marine Agar are regular, convex, with regular border and non-pigmented. May present opaque and transparent colony variants. Cells do not accumulate polyhydroxybutyrate (PHB). Aerobic chemoorganotrophic bacterium, unable to ferment carbohydrates. Oxidase and catalase positive. Grows from 5–42 ºC, but not at 45 ºC (optimum 26–30 ºC) and from 1 to 12% salinity but not at 0.5 or lower, or 18% or higher. Values of pH supporting growth are 6.0–9.0, with optimal growth at 7–8. Reduces nitrates to nitrites but not to N_2_. It does not produce indole. Hydrolyzes gelatin but not urea, esculin, starch, casein, Tween 80 or alginate. Does not assimilate glucose, arabinose, mannose, mannitol, N-acetyl-glucosamine, maltose, gluconate, caprate, adipate, malate, citrate or phenylacetate in API 20NE. Negative for arginine dihydrolase, lysine decarboxylase and ornithine decarboxylase activities. Unable to grow on any of the following sole carbon and energy sources: d-xylose, l-arabinose, d-mannose, d-cellobiose, sucrose, d-melibiose, lactose, d-sorbitol, d-gluconate, d-glucuronate, 2-oxoglutarate, 3-hydroxybutyrate and putrescine. Unable to oxidize or ferment any carbohydrate in API 50CH/E strips. Positive for alkaline phosphatase and leucine arylamidase activities but negative for estearase C4, estearase lipase C8, lipase C14, valine arylamidase, cystine arylamidase, trypsin, α-chymotrypsin, acidic phosphatase, naphthol-AS-BI-phosphohydrolase, α-galactosidase, β-galactosidase, β-glucuronidase, α-glucosidase, β-glucosidase, N-acetyl β-glucosaminidase, α-mannosidase and α-fucosidase in API ZYM.

Major cellular fatty acids are C_15:0_ iso, Summed Feature 9 (C_17:1_ iso *ω9c*/C_16:0_ 10-methyl) and C_17:0_ iso. G + C molar content is 49.5 mol%.

Type strain is CECT 9734^ T^ (= LUBLD50 7a^T^ = LMG 31044^ T^), it was isolated from liver of a cultured seabass (*Dicentrarchus labrax)*. The accession numbers of 16S rRNA gene sequence and draft genome of the type strain are MN809182 and CACRZB01, respectively. The type strain genome size is 2.5 Mbp.

## Supplementary Information

Below is the link to the electronic supplementary material.

Supplementary file1 (PDF 351 KB)
