# Supplementary material for: Pseudidiomarina piscicola sp. nov., isolated from cultured European seabass, Dicenthrarchus labrax
Source: Arch Microbiol. 2020 Dec 7;203(4):1293–8. doi: 10.1007/s00203-020-02131-3 (PMC8055624; doi:10.1007/s00203-020-02131-3)

Pseudidiomarina piscicola sp. nov., isolated from cultured European seabass, *Dicenthrarchus labrax*

Archives of Microbiology

M. Carmen Macián, Teresa Lucena, David R. Arahal, María A. Ruvira, Rosa Aznar and María J. Pujalte

Departamento de Microbiología y Ecología and Colección Española de Cultivos Tipo (CECT). Universitat de València. SPAIN.

Supplementary Table S1. Cellular fatty acid composition (%) of *Pseudidiomarina piscicola* strain CECT 9734^T. Major components (> 10 %) are in bold; tr: less than 1%.

Fatty acid	%
10:0	tr
11:0 iso	1.5
10:0 3OH	tr
12:0	tr
11:0 iso 3OH	5.1
13:0 iso	1.4
12:0 3OH	1.1
14:0	tr
13:0 iso 3OH	3.9
15:1 iso F	2.5
**15:0 iso**	**21.6**
15:0 anteiso	tr
16:0 iso	tr
16:1 *ω*9c	tr
Sum In Feature 3	6.9
16:0	9.0
**Sum In Feature 9**	**18.5**
**17:0 iso**	**14.8**
17:0 anteiso	tr
17:1 *ω*8c	tr
17:0 cyclo	tr
17:0	tr
18:1 *ω*9c	1.2
Sum In Feature 8	4.9
18:0	2.3
19:0 iso	tr
20:1 *ω*7c	tr

Sum In Feature 3: $C_{16:1}$ *ω*7c/*ω*6c; Sum In Feature 9: $C_{17:1}$ iso *ω9c*/$C_{16:0}$ 10-methyl; Sum In Feature 8: $C_{18:1}$ *ω*7c/*ω*6c

Supplementary Figure S1. Cell morphology of strain CECT 9734^{T} as determined by optical microscopy. Picture shows rod-shaped cells motile by a single polar flagellum observed after staining with Ryu dye (Heimbrook ME, Wang WL, Campbell G (1989) Staining bacterial flagella easily. J Clin Microbiol 27: 2612-2615).

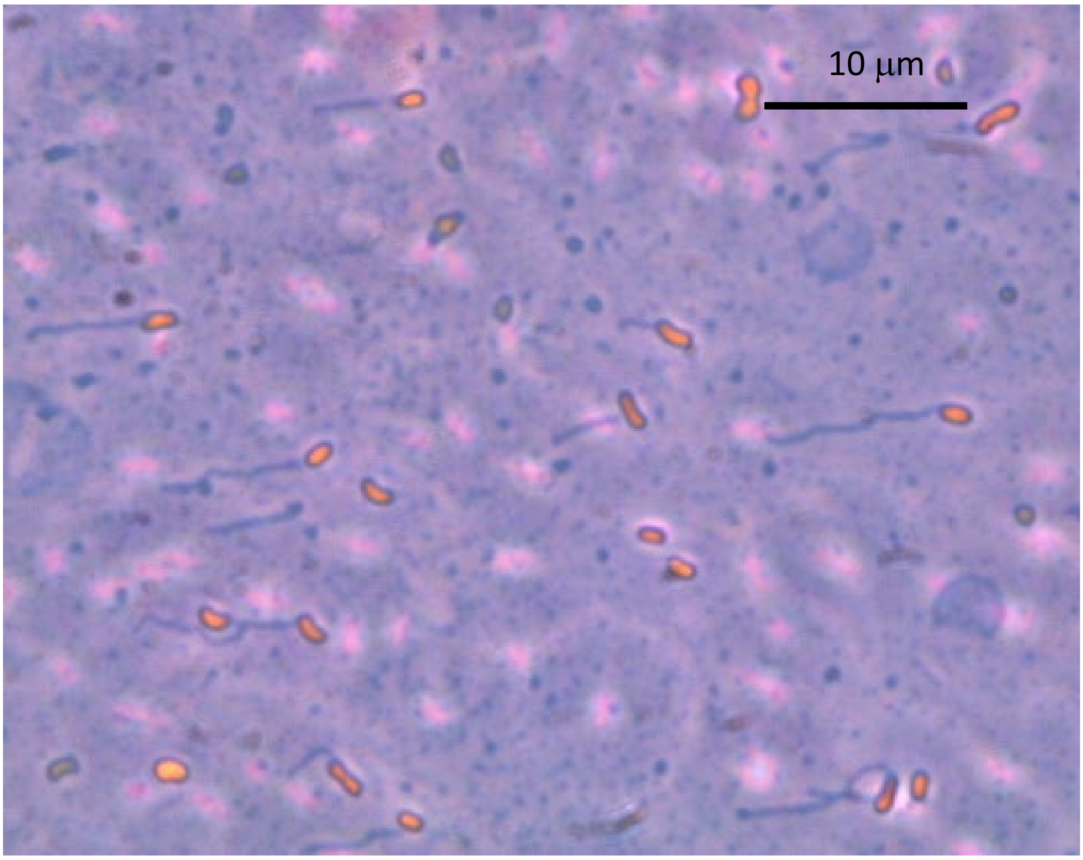

Supplementary Figure S2. Phylogenomic tree generated with UBCG (Na et al., 2018) by using nucleotide sequences. The numbers at the nodes indicate the Gene Support Index (GSI, maximal value is 92). Genome accession numbers are indicated in parentheses. Bar, 0.05 substitutions per position.

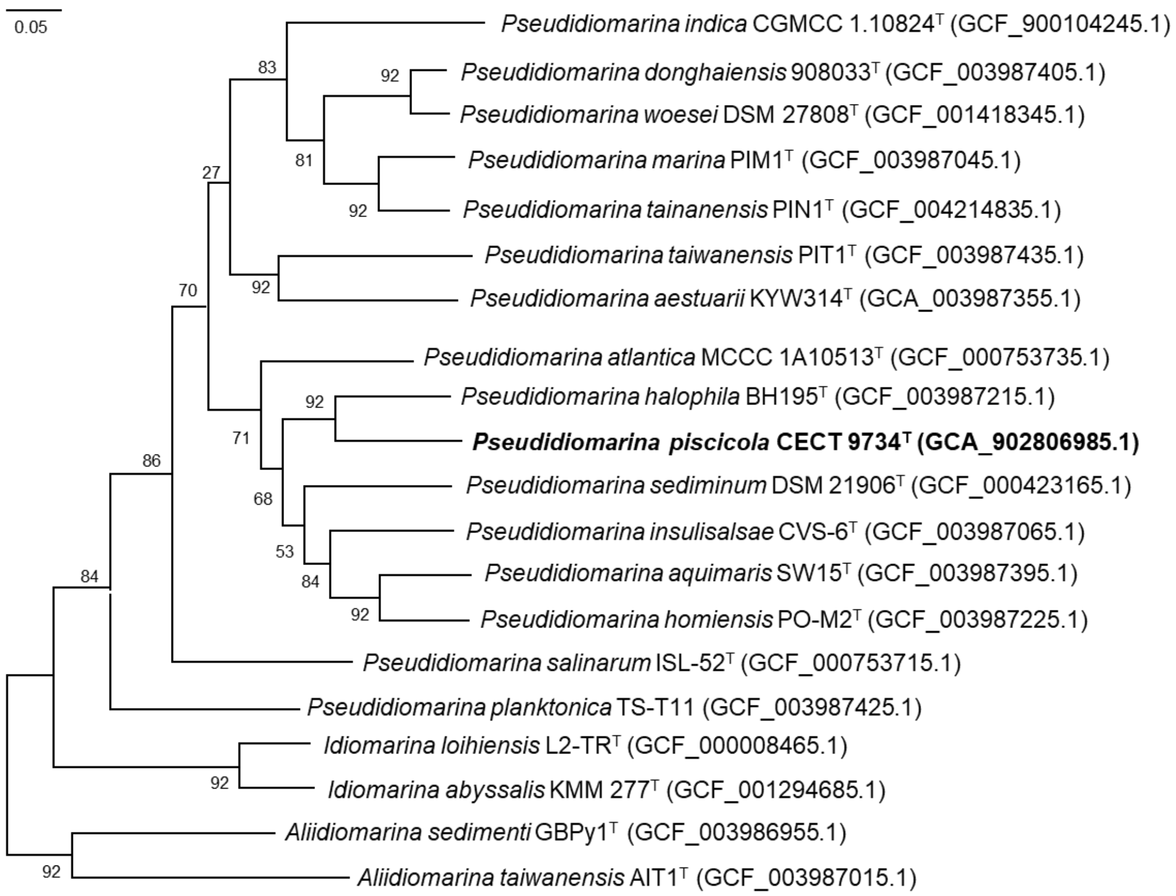

Supplement: Supplementary file 1 — Supplementary file1 (PDF 351 KB) [file 203_2020_2131_MOESM1_ESM.pdf]
